# Seroprevalence of Human Fasciolosis in a New-Emerging Focus of Fasciolosis in Yasuj District, Southwest of Iran

**Published:** 2012

**Authors:** B Sarkari, N Ghobakhloo, AA Moshfea, O Eilami

**Affiliations:** 1 Center for Basic Researches in Infectious Diseases, Shiraz University of Medical Sciences, Shiraz, Iran; 2 Department of Parasitology and Mycology, School of Medicine, Shiraz University of Medical Sciences, Shiraz, Iran; 3 Department of Parasitology, School of Medicine, Yasuj University of Medical Sciences, Yasuj, Iran; 4 Department of Infectious Diseases, School of Medicine, Yasuj University of Medical Sciences, Yasuj, Iran

**Keywords:** Seroepidemiology, Fasciolosis, New emergence, Iran

## Abstract

**Background:**

Fasciolosis is an important health and veterinary problem in Iran. The epidemiological pattern of disease has been changed markedly in recent years and there are regions that have potent capacity to be new focus of the disease. One of these areas is Yasuj district in southwest of Iran where animal fasciolosis has been quite common. The current study was conducted to determine the seroprevalence of human fasciolosis in this area and to reveal the epidemiological factors associated with the spreading of the disease in this region.

**Methods:**

One thousand blood samples were randomly collected from five villages in Yasuj district. ELISA, using *Fasciola* somatic antigen (SA), was carried out to detect anti *Fasciola* antibodies in the collected sera.

**Results:**

Anti-*Fasciola* antibodies were detected in serum of 18(1.86%) individuals by ELISA. Out of 18 seropositive people, 9 (0.9) were female and 9 (0.9%) were male. Most of people (99.8%) had a history of consuming wild freshwater plants mainly *Nasturtium microphyllum* (local name Bakaloo) and/or *Mentha logifolia* (local name Pooneh). No significant correlation was found between seropositivity to fasciolosis and sex, age, history of consumption of green leafy aquatic plants whereas correlation between seropositivity and abdominal pain was significant (*P*< 0.05).

**Conclusion:**

Results of this study showed that the seroprevalence rate of human fasciolosis in Yasuj district is relatively high and this area can be considered as a new emerging focus of the disease in Iran.

## Introduction

Fasciolosis is a zoonotic disease caused by hepatic trematodes *Fasciola hepatica* and *Fasciola gigantica*. Human become infected by eating aquatic plants or by drinking water contaminated with metacercariae (1–2).

Although fasciolosis has usually been considered as a major veterinary importance, human fasciolosis has recently been regarded as a serious health problem in many countries in the world (1, 3). Currently the epidemiological pattern of disease has been changed and the disease is emerging or re-emerging in many areas of the world (4).

In Iran, fasciolosis is common among domestic animals in most parts of the country and its prevalence reaches up to 50% in some areas (5–7). Human fasciolosis is a matter of concern in provinces situated along the shore of the Caspian littoral; especially in Gilan Province (6). This province has experienced two waves of the fasciolosis epidemics. The first wave was begun in 1987 when an outbreak in Gilan affected more than 10,000 people. The second wave of the epidemic began within 10 years later where several thousand people were infected. Reports of several hundred cases of human fasciolosis between two outbreaks and afterward show that Gilan Province has become an endemic area for human fasciolosis in Iran. At least 17000 human infections have been reported in Gilan Province since 1989 (6).

Cases of human fasciolosis have been reported from other parts of the country as well. One of these area is Kohgiluyeh and Boyerahmad Province located in southwest of the country. Fasciolosis is quite common in sheep and cattle in this region. In a study conducted by Moshfea et al., about fasciolosis in slaughtered animals in Yasuj, 12.5% of cattle, 11.75% of sheep and 7.16% of goats were infected by *Fasciola* spp. (7).

High prevalence of fasciolosis in animals in this region ensure the distribution of the infection, and food habitant of people for eating the raw water plants favor the endemicity of human fasciolosis in this region. Considering the aforementioned criteria, human cases of fasciolosis might be present in this region. Moreover, human cases of fasciolosis in the region have been claimed by some physicians but no confirmatory published data are available about this. This study was conducted to determine the seroprevalence of human fasciolosis in this area and to reveal the epidemiological factors associated with the spreading of the disease in the region.

## Materials and Methods

### The study area

**Fig. 1:**
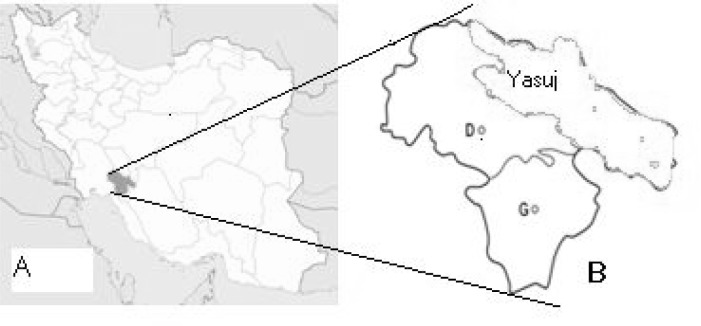
Map of Iran (A) and the Yasuj district (green) in Kohgiluyeh and Boyerahmad (B) province

**Fig. 2:**
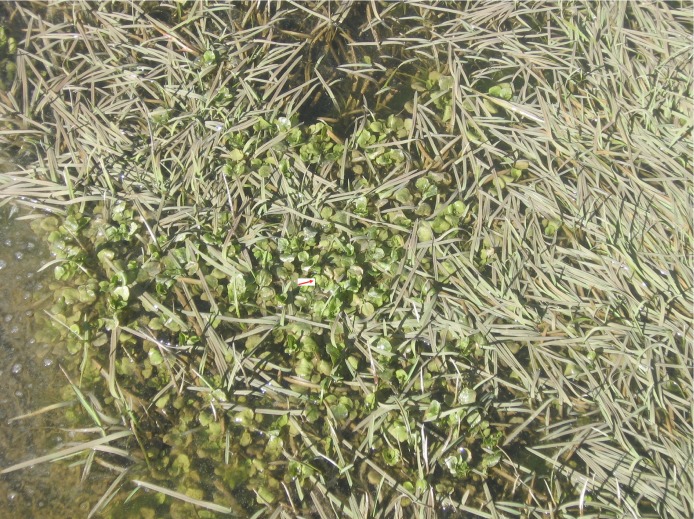
Wild freshwater plant *Nasturtium microphyllum* (Local name Bakaloo) commonly used in Yasuj district

The study was carried out in Yasuj district, located in the southwest of Iran (300 40° N 51°35°E). The district covers an area about 15563 sq km (Fig. 1). The vegetation of the area includes wild pistachio and tulips and most of the area in the region are covered with oak forests. Inhabitants of the district live mainly on animal farming and gardening. The traditional wild freshwater plants such as *Nasturtium microphyllum* (local name Bakaloo) and *Mentha logifolia* (local name, Pooneh) are consumed by local people in this area (Fig. 2). People usually use *Nasturtium microphyllum* as a raw vegetable with their food.

### Serum samples

Subjects of this study were from five villages (Tangari, Sargachineh, Koskolya, Cheshmehpahn and Jahanabad) in Yasuj district (Fig. 1). Blood samples were collected from inhabitants of each villages based on the population of each village.

Ethical approval of the study was given by the Ethic Committee of Shiraz University of Medical Sciences and consent was obtained from the participants or their guardians. Venous blood specimens of 5 ml were obtained from 1000 participants. Blood were centrifuged (2500 g) for 10 min to obtain serum. Collected sera were stored at −20°C until use. All participants completed a questionnaire containing data such as, age, gender, consuming aquatic vegetables, type of vegetables and questions about general (nausea, vomiting, fever, and weight loss) and gastrointestinal symptoms.

### Preparation of Fasciola somatic antigen (SA)

Living adult *Fasciola hepatica* worms were obtained from the bile ducts of infected sheep collected from Shiraz industrial slaughterhouse. The worms were washed, five times, with sterile PBS, to remove blood and bile remains. Somatic antigen was prepared by homogenizing worm in PBS using electrical homogenizer, followed by sonication in cold condition and centrifugation at 15000 g at 4°C for 30 min. The supernatant were collected as somatic antigen. The protein content of sample was estimated by Bradford method and the antigen was kept at −20°C until use.

### Enzyme Linked Immunosorbent Assay

ELISA, using *Fasciola* somatic antigen (SA), was carried out in flat bottom 96-well microplates (Nunc, Nagle, Nunc, International, Roskilde, Denmark). The plates were coated with 5 µg/ml of somatic antigen (100 µg/well) in coating buffer (0.05 M carbonate-bicarbonate buffer, pH 9.6) and incubated at 4° C overnight. Plates were washed 5 times in phosphate-buffered saline-Tween 20 (PBST, pH 7.4 containing 0.05% Tween 20). Blocking was performed with 3% skim milk in PBST for 1.5 hours. Plates were washed as before and 100 µl of serum samples (1/100 dilution in PBST) was applied to the plates and incubated for 1.5 hour at room temperature. The plates were washed as before and 100 µl aliquots of horseradish peroxidase conjugated polyclonal antibody against human immunoglobulin (Sigma) at a 1/2500 dilution in PBST was added to the plates and incubated for 1 hour at room temperature. After washing as before, the plates were incubated with chromogen/substrate (100 µl/well of OPD, 0.025% H_2_O_2_ in 0.1 M citrate buffer, pH 5) and after 30 min the absorbance at 490 nm was measured using a microplate reader.

Positive controls (5 sera samples from confirmed fasciolosis patients) were kindly provided by Dr Rokni from School of Health at Tehran University of Medical Sciences and negative samples were taken from healthy individuals from non-endemic area who had no history of fasciolosis. The cut off point was set at 3SD above the mean of negative controls.

### Statistical analysis

SPSS (Statistical Package for Social Sciences) for windows version 13 was used for analysis of the data. Chi-square test was used to determine the significance difference between positive seroprevalence rates and epidemiological factors associated with fasciolosis.

## Results

In this study, 1000 serum samples were obtained from Yasuj district in Southwest of Iran. Subjects were consisted of 682 (68.2%) females and 318 (31.8%) males. Their ages ranged between 7 to 91 years old. Most of people (99.8%) had a history of consuming wild freshwater plants mainly *Nasturtium microphyllum* (local name, Bakaloo) and/or *Mentha logifolia* (local name, Pooneh).

Consuming of *Nasturtium microphyllum* was much common (85%) among the participant in comparison with *Mentha logifolia*. While 847 (84.7%) of subjects had no complain, others complained about clinical manifestations such as abdominal pain, headache, nausea or jaundice.

Anti-*Fasciola* antibodies were detected in 18 (1.8%) of the participants by ELISA. Out of 18 seropositive people, by chance, nine (0.9%) were female and 9 (0.9%) were male. No significant correlation was found between sex, and seropositivity to fasciolosis. Considering the age of subjects, the highest prevalence of fasciolosis (3.7%) was found in the 41–50-year old age group. However, the difference between the age of subjects and the presence of antibody against fasciolosis was not statistically significant (*P*>0.05). Table 1 shows the seroprevalence of fasciolosis in different age groups.

**Table 1:** Seroprevalence of human fasciolosis according to age groups in Yasuj district

Age (yr)	groups	No. of cases	No. of positive cases	Percent
1–10		83	2	2.4
11–20		273	4	1.46
21–30		269	5	1.85
31–40		175	4	2.28
41–50		81	3	3.7
>51		119	0	0
Total		1000	18	1.8

Findings of this study show that all of seropositive subjects had a history of consuming green leafy vegetables. However, the differences between seronegative and seropositive people was not statistically significant (*P*>0.05). Considering the clinical signs and symptoms of the subjects, there was a significant difference (*P*< 0.05) between seropositivity to fasciolosis and having at least one sign or symptom (mainly abdominal pain) related to fasciolosis.

## Discussion

Fasciolosis is an important health, veterinary and economic problem in many countries of the world including Iran (3, 6). The epidemiological pattern of disease has been changed markedly in recent years and there are region that has a potent capacity to be a new focus of the disease (1, 4).

Animal fasciolosis is quite common in Iran (5–6). In a study conducted by Eslami et al., in north of the country, fecal samples of 32% of sheep, 32.1% of cattle, 17% of buffaloes, 50% of horses were found to be infected with *Fasciola* egg (8).

Along with animal fasciolosis, human fasciolosis is a major health concern in few provinces of the country including Gilan.

While the prevalence of fasciolosis among domestic animals in the southern part of the country is the same or even higher than (up to 80% in buffalo) those in the northern part, the number of human fasciolosis reported is significantly higher in the Northern provinces (9–11).

Iran has been the site of what is called the biggest outbreak of human fasciolosis in the world during 1987–97.

Nowadays human cases are being reported from different part of Iran, although the focuses of human fasciolosis are in the north of the country (5). Moreover new foci of the disease seems to be emerging and one of these focus is in southwest of the country, in Yasuj district. High prevalence of animal fasciolosis, unconfirmed reports of human cases from this area in the last two years and lack of information about the epidemiology of this disease justified the performing of the current study as the first study on seroepidemiology of human fasciolosis in this area.

Findings of this study revealed that 1.8% of the populations are seropositive for fasciolosis. This means that this province is a mesoendemic area for human fasciolosis according to the classification by Mas-coma (1). High incidence of human fasciolosis in this area can be explained by the fact that people in this region raised herd of sheep, cattle and goat, are dependent on agriculture for their income, and consume large amount of traditional aquatic plant mainly *Nasturtium microphyllum* and *Mentha logifolia*. *Nasturtium microphyllum* is very popular kind of wild plants, which is eaten raw, in some popular local dishes.

Our finding about the rate of fasciolosis is almost in harmony with those seroprevalence studies, which have been carried out in the region. In a seroprevalence study conducted by Turhan et al., in Antalya in Turkey, 3% of participants were seropositive for fasciolosis and the area defined as mesoendemic for human fasciolosis (12).

In another study performed by Ozturhan et al., in Mersin province in Turkey, 0.79% of the participants were found to be seropositive for *Fasciola hepatica* and the prevalence of fasciolosis was considered as hypoendemic in the region (13).

In our study, the rate of seropositivity for fasciolosis was the same in both genders. This means that both males and females are equally prone to acquiring the infection. This is in consistent with some of those reports from other fasciolosis endemic areas (4–6, 9–10). However, in Andean countries and Egypt the prevalence of fasciolosis in females appears to be significantly higher than in males (14–16).

Correlation between age and fasciolosis was not significant in our study. This again is in agreement with results of other studies, which have been done in other parts of Iran, in Africa and South Asia (2, 5, 14–15).

Aquatic plant consumption has already been proven as one of the most important risk factor in acquiring *Fasciola* infection (2, 17–18). In our study, there were no significant difference between consumption of aquatic herb and seropositivity for fasciolosis. This is mainly because almost all of people in this region are using green aquatic vegetable, especially *Nasturtium microphyllum* (local name, Bakaloo).

Fasciolosis may cause a wide variety of clinical sings and symptoms ranging from asymptomatic infection to sever disease including anemia. In our survey, we determined some nonspecific symptoms such as abdominal pain, allergy, and headache in seropositive individuals. Furthermore, correlation between the seropositivity to fasciolosis and having at least one of sign or symptoms (mainly abdominal pain) related to fasciolosis was significant.

In conclusion, findings of this study showed that the seroprevalence rate of fasciolosis in Yasuj district in Kohgiluyeh and Boyerahmad province is relatively high and this area can be considered as a new-emerging focus of the disease in Iran. However, we believe that further studies including finding the source of the disease are needed to elucidate appropriate situation of human fasciolosis in this region.
